# The EU-Emotion Voice Database

**DOI:** 10.3758/s13428-018-1048-1

**Published:** 2018-04-30

**Authors:** Amandine Lassalle, Delia Pigat, Helen O’Reilly, Steve Berggen, Shimrit Fridenson-Hayo, Shahar Tal, Sigrid Elfström, Anna Råde, Ofer Golan, Sven Bölte, Simon Baron-Cohen, Daniel Lundqvist

**Affiliations:** 1 Autism Research Centre, Department of Psychiatry, University of Cambridge, Cambridge, UK; 2 Brain & Cognition, Department of Psychology, University of Amsterdam, Amsterdam, The Netherlands; 3 Institute of Psychiatry, King’s College London, London, UK; 4 Institute for Women’s Health, University College London, London, UK; 5 KIND, Department of Women’s and Children’s Health, Karolinska Institutet, Stockholm, Sweden; 6 Center of Psychiatry Research, Stockholm County Council, Stockholm, Sweden; 7 Department of Psychology, Bar-Ilan University, Ramat Gan, Israel; 8 CLASS Clinic, Cambridgeshire and Peterborough NHS Foundation Trust, Cambridge, UK; 9 NatMEG, Department of Clinical Neuroscience, Karolinska Institutet, Stockholm, Sweden

**Keywords:** Voice stimuli set, Multisite validation, Emotion perception

## Abstract

In this study, we report the validation results of the EU-Emotion Voice Database, an emotional voice database available for scientific use, containing a total of 2,159 validated emotional voice stimuli. The EU-Emotion voice stimuli consist of audio-recordings of 54 actors, each uttering sentences with the intention of conveying 20 different emotional states (plus neutral). The database is organized in three separate emotional voice stimulus sets in three different languages (British English, Swedish, and Hebrew). These three sets were independently validated by large pools of participants in the UK, Sweden, and Israel. Participants’ validation of the stimuli included emotion categorization accuracy and ratings of emotional valence, intensity, and arousal. Here we report the validation results for the emotional voice stimuli from each site and provide validation data to download as a supplement, so as to make these data available to the scientific community. The EU-Emotion Voice Database is part of the EU-Emotion Stimulus Set, which in addition contains stimuli of emotions expressed in the visual modality (by facial expression, body language, and social scene) and is freely available to use for academic research purposes.

**Electronic supplementary material:**

The online version of this article (10.3758/s13428-018-1048-1) contains supplementary material, which is available to authorized users.

## Background

The tone of voice, or *prosody*, of others is an important cue to understand their affective states. During a social interaction, prosody is key to accurately determining the emotion that others are experiencing (Banse & Scherer, 1996). Even very young infants are capable of recognizing the different intonations of their mothers (Fernald, 1989; Fernald & Morikawa, 1993). Clinical conditions, however, can alter both the production and the recognition of intonation. For instance, atypical emotional prosody has long been considered a hallmark of autism, a neurodevelopmental condition marked by deficits in social communication and interaction (Asperger, 1944; Kanner, 1943). Abnormal tone of voice was noted as the primary contributor to the perceived oddness of people with autism during social interaction (Paul et al., 2005; Van Bourgondien & Woods, 1992), and thus puts them at risk of social exclusion. In addition, many studies have reported a deficit in emotional prosody perception in people with autism (Globerson, Amir, Kishon-Rabin, & Golan, 2015; Golan, Baron-Cohen, Hill, & Rutherford, 2007; Rutherford, Baron-Cohen, & Wheelwright, 2002), which could explain some of their social difficulties. Learning how to recognize and apply emotional prosody is hence a common challenge for people with autism, and an important skill to train in order to increase their chance of social inclusion.

### Training recognition and production of prosody in autism

This study is part of a larger project in which the authors (and additional collaborators) developed and evaluated an educational online game to train autistic children 5–10 years old to recognize and produce emotional prosody.

In this article, we report the production and validation of the *EU-Emotion Voice Database*, a unique and large set of emotional vocal stimuli that were used as training material for the activities aimed at helping children with autism recognize and express emotions in the vocal modality. The EU-Emotion Voice Database also served in the development of a *voice analyzer* (Marchi et al., 2015)*.* This voice analyzer was trained with the EU-Emotion voice stimuli—via machine learning—to become able to discern the properties of an emotional voice necessary for a particular emotion to be identified by a human listener. Finally, we used the EU-Emotion Voice Database to provide a pool of validated emotional voice stimuli for a psychology experiment investigating differences in emotional prosody recognition between children with and without autism in the UK, Sweden, and Israel (Fridenson-Hayo et al., 2016).

### The EU-Emotion Voice Database

The features of the EU-Emotion Voice Database differ from those of other existing emotional voice databases (see Table 1 for an overview of published emotional voice databases) in several ways. First, it includes emotional vocal stimuli for 20 different emotions (plus neutral), whereas most databases are limited to fewer emotions (Bänziger, Mortillaro, & Scherer, 2012; Hawk, Van Kleef, Fischer, & Van Der Schalk, 2009). Most previous emotional voice databases have included at least some of the six basic emotions (fear, anger, surprise, sadness, happiness, and disgust), because those emotions are thought to reflect innate and culturally universal emotion categories (Ekman & Friesen, 1971). However, complex/subtle emotions, which may be culturally dependent and mastered later in life, have rarely been included to any substantial extent in previous emotional voice databases (see Table 1). The presence of several subtle/complex emotions in this database, in addition to the basic six, is hence novel, unique, and important to the study of emotions, as it permits investigation of emotion recognition abilities for complex/subtle emotions expressed through the voice alone.

**Table 1:** Summary of the main characteristics of a selection of published emotional databases from 1996 to 2016

Authors	Year	Emotions	Language	Stimulus Type	Actors	Judges	Ratings
Banse & Scherer	1996	14 (hot anger, cold anger, panic fear, anxiety, despair, sadness, elation, happiness, interest, boredom, shame, pride, disgust, and contempt)	NA	Meaningless sentences composed of phonemes from Indo-European languages but resembling speech	12 actors (6 females)	12	accuracy
Polzin & Waibel	1998	4 (happiness, sadness, anger, fear)	1 (English)	50 sentences per emotion	5 acting students	“subjects”	accuracy
Pereira	2000	4 (happiness, sadness, hot anger, cold anger + neutral)	1 (English)	40 sentences per emotion	2 actors	31	arousal, pleasure, and power
Scherer	2000	5 (fear, disgust, joy, sadness, anger)	NA	2 sentences in an artificial language made by a phonetician	4 actors	20	accuracy
Abelin & Allwood	2000	8 (joy, surprise, sadness, fear, shyness, anger, dominance, disgust)	1 (Swedish)	1 sentence with neutral content	1 male speaker	35	accuracy (free choice)
Niimi, Kasamatsu, Nishimoto, & Araki	2001	3 (anger, sadness, joy)	1 (Japanese)	Vowel–consonant–vowel (VCV) segments from 400 sentences with neutral content	1 male speaker	12	accuracy
Schröder	2003	10 (admiration, threat, disgust, elation, boredom, relief, startle, worry, contempt, hot anger)	1 (German)	nonspeech utterances	6 speakers (3 females)	20	accuracy, arousal, valence, control
Scherer & Ellgring	2007	14 (hot anger, cold anger, panic fear, anxiety, despair, sadness, elation, happiness, interest, boredom, shame, pride, disgust, contempt)	NA	2 sentences in artificial language made by a phonetician	12 actors (6 females)	NA	NA
Belin, Fillion-Bilodeau, & Gosselin	2008	8 (anger, disgust, fear, pain, sadness, surprise, happiness, pleasure+neutral)	NA	1 short emotional interjection ("ah") per emotion	10 actors (5 females)	30	accuracy, valence, arousal, intensity
Hawk, Van Kleef, Fischer, & Van Der Schalk	2009	9 (anger, contempt, disgust, embarrassment, fear, joy, pride, sadness, surprise, + neutral)	1 (Dutch)	nonlinguistic affect vocalization and speech-embedded expressions of emotions	8 acting students (4 females)	121	accuracy
Pell, Paulmann, Dara, Alasseri, & Kotz	2009	6 (joy, sadness, anger, fear, disgust, pleasant surprise + neutral)	4 (Spanish, English, German, Arabic)	pseudo-utterances (“nonsense speech”)	4 native speakers with “amateur experience in acting or public speaking” (2 females) per language	61	accuracy
Bänzinger, Mortillaro, & Scherer	2012	18 (joy, amusement, pride, pleasure, relief, interest, panic fear, despair, cold anger, anxiety, sadness, disgust, contempt, shame, admiration, tenderness, surprise)	NA	2 pseudo-speech sentences and a nonverbal utterance	10 actors (5 females)	20	accuracy, intensity
Liu & Pell	2012	6 (joy, sadness, anger, fear, disgust, pleasant surprise + neutral)	1 (Mandarin Chinese)	pseudo-utterances (“nonsense speech”)	4 native speakers (2 females)	24	accuracy, intensity

Second, the database contains emotional voice stimuli, portrayed by a total of 54 actors across a wide age span and across three languages, resulting in a total of more than 2,000 validated stimuli. The inclusion of emotional voice stimuli in three different languages (British English, Swedish, and Hebrew) is another novel aspect of the EU-Emotion Voice Database, as most previous emotion voice databases had stimuli in one language only (see Table 1). Thus, the EU-Emotion Voice Stimuli could be useful for studying emotion perception from vocal cues cross-culturally as a way to shed light on the aspects of emotional prosody that are culture specific, and those that are universal.

Naturally, when creating a large database including a wide range of emotional expressions with the purpose of training children with autism, it is crucial to assess the degree with which each stimulus conveys the intended emotion. Therefore, all the stimuli in the EU-Emotion Voice Database were validated by typically developing adults for emotion recognition accuracy and perceived emotional valence, intensity, and arousal. (For similar validation approaches on vocal emotional stimuli, see Schröder, 2003, and Belin, Fillion-Bilodeau, & Gosselin, 2008.) Here we report the validation results of those stimuli, so as to make them available to the wider scientific community.

Finally, although the EU-Emotion Voice Database is a large and unique pool of stimuli in its own right, it is part of a larger emotional stimulus database, the *EU-Emotion Stimulus Set*.^1^ This includes emotional stimuli expressed in the visual modality (facial expressions, body language, and social scenes^2^; see O’Reilly et al., 2016). Only one previous database has contained both audio and visual emotional stimuli (Bänziger et al., 2012), but it does not include social-scene stimuli that provide the contextual cues that are potentially important to recognize certain complex emotions.

## Method

### Voice stimuli creation

#### Actors

Three sets of healthy actors (*N* = 18 per site, nine females) were recruited to express the different emotions.^3^ The actors in each set were either native speakers of British English, Swedish, or Hebrew. Their age ranged from 10 to 70 years in the UK, and 9 to 67 years in Sweden and 11 to 72 in Israel (see Table 2). The actors were recruited from professional acting agencies or drama schools within the three countries. Trained researchers from each site guided the actors through their performance.

**Table 2:** Demographic tables for (a) the British actors, (b) the Swedish actors, and (c) the Israeli actors

a)					b)					c)		
UK					Sweden					Israel		
Actor Code	Age	Gender	Script	Mean CCR	Actor Code	Age	Gender	Script	Mean CCR	Actor Code	Age	Gender
A	19	Female	A	48%	AF04A1	18	Female	A	28%	Ai	20	Female
B	37	Female	B	39%	AF02B1	27	Female	B	44%	Bi	36	Female
C	31	Male	A	36%	AF01A1	39	Female	A	40%	Ci	35	Male
D	27	Female	B	47%	AF05B2	44	Female	B	48%	Di	29	Female
E	70	Female	A	36%	AF03A2	67	Female	A	34%	Ei	72	Female
G	15	Female	B	39%	AM01B1	18	Male	B	NA*	Fi	12	Female
H	62	Male	A	28%	AM04B2	31	Male	B	46%	Gi	15	Female
K	30	Male	A	37%	AM02A1	45	Male	A	33%	Hi	68	Male
M	37	Male	B	45%	AM03A2	65	Male	A	NA*	Ii	11	Male
N	42	Female	A	37%	YF03B1	8	Female	B	27%	Ji	12	Male
O	21	Female	A	37%	YF02A1	11	Female	A	38%	Ki	35	Male
F	10	Female	B	32%	YF01B2	12	Female	B	37%	Li	11	Male
I	11	Male	B	42%	YF04A2	14	Female	A	30%	Mi	37	Male
J	12	Male	B	42%	YM01B1	9	Male	B	35%	Ni	42	Female
L	12	Male	B	37%	YM05B2	9	Male	B	52%	Oi	19	Female
P	12	Male	B	44%	YM04B1	13	Male	B	44%	Pi	13	Male
Q	10	Female	A	36%	YM02A2	14	Male	A	28%	Qi	12	Female
S	11	Male	B	35%	YM03A1	14	Male	A	26%	Ri	11	Male

Each actor portrayed a subset of emotions (either set A or set B, each including ten emotional states) such that each emotion is portrayed by a subset of actors. The two sets were randomly distributed among the actors as follows: *UK*—Script A: 5 females, 3 males, 1 child, 7 adults (18+); Script B: 4 females, 6 males, 7 children, 3 adults (18+). *Sweden*—Script A: 5 females, 4 males, 4 children, 5 adults (18+); Script B: 5 males, 4 females, 5 children, 4 adults (18+). *Israel*—Script A: 5 females, 4 males, 4 child, 5 adults (18+); Script B: 4 females, 5 males, 4 children, 5 adults (18+). CCR, chance-corrected recognition rate. *None of the stimuli were kept in the database for this actor, due to poor quality

#### Emotions

The emotional voices in the EU-Emotion Stimulus Set include 20 emotional states (*afraid*, *angry*, *ashamed*, *bored*, *disappointed*, *disgusted*, *excited*, *frustrated*, *happy*, *hurt*, *interested*, *jealous*,^4^*joking*, *kind*, *proud*, *sad*, *sneaky*,^5^*surprised*, *unfriendly*, *worried*) and the *neutral* state. These 20 emotional states were selected from originally 27 by autism experts (*n* = 47) and parents of children with autism (*n* = 88), who perceived them as the most important states for social interaction (see Lundqvist et al., 2013).

#### Scripts

There were two different scripts for the sentences to be read by the actors (see the supplementary materials, Table A). These voice scripts were first written in English and then translated to Swedish and Hebrew, using back-translation. Each of those two scripts contained both semantically neutral and semantically emotional sentences for the ten emotional states. The sentences contained two to ten words apiece (mean = 4.64, *SD* = 1.14). Each actor was assigned to one script (i.e., one set of emotional state), and thus produced both semantically neutral and semantically emotional sentences. The semantically neutral sentences were produced for all different emotions, but the semantically emotional sentences only in the compatible emotion. For each sentence, the actors produced three items or exemplars. The same protocol was used across the three sites.

#### Recordings

In the UK and in Sweden, the six basic emotions (anger, disgust, fear, happiness, sadness, and surprise) were portrayed at high and low intensity. The other 14 complex emotional states were expressed at a high intensity only. The following example instruction was given to help guide the intensity of expression across all modalities: “High Intensity—In this situation, you are quite angry, not a little angry, not very angry, but quite and unmistakably angry.” In Israel, the actors were asked to express the emotions naturally, with no separate expressions of high and low intensity (and no explicit instruction). Each actor portrayed only a subset of the ten emotional states (three basic and seven complex). The ten emotional states portrayed by each actor depended on the script they had been assigned (see the supplementary materials, Table A), and the two scripts were enacted by equal numbers of actors with comparable distributions of gender and age (Table 2). The members of the research team provided feedback throughout to guide the actors’ performances. A total of 4,781 voice stimuli (British English *k* = 1,569, Swedish *k* = 1,574, Hebrew *k* = 1,638) were recorded in a soundproof studio at each site.

### Voice stimuli validation process

#### Stimulus selection

##### UK

The actors recorded each script three times, and the best portrayal the actor made of that script was selected by the recording company, under the supervision of a trained researcher, to go through for validation. This procedure resulted in the discarding of 56% of the originally recorded stimuli and in the selection of 695 stimuli.

##### Sweden

A large proportion of the stimuli (36%) were also discarded in the Swedish sample, due to low acting quality. Similar to the UK, the best portrayals that an actor made of each script was kept, and poor-quality portrayals were discarded. The selection of the stimuli was conducted by the two experienced psychologists involved in the recording of all voice stimuli. This resulted in the selection of 1,011 stimuli.

##### Israel

All recorded stimuli were judged by three members of the Israeli research team. Only those unanimously judged as clearly depicting the target emotion were kept for the validation procedure (72%). This resulted in the selection of 453 stimuli.

#### Survey design

The survey structure was first developed in British English and then translated into Swedish and Hebrew (using back-translation) by two native speakers for each language who were also fluent in British English. A total of 84 surveys were distributed (20 in the UK, 30 in Sweden, and 34 in Israel). The online surveys were constructed in such a way that the emotional states were evenly distributed across surveys (to ensure that each emotion category was represented in each survey) and included 34–35 voice stimuli in Sweden and the UK, and 16–18 stimuli in Israel. Each stimulus appeared in one survey only. Each survey took approximately 30 min to complete, and each survey responder responded to only one survey. For each stimulus, survey responders were asked (1) to discriminate the emotion expressed by the voice among six possible choices and (2) to assess the expressed emotion on arousal, valence, and intensity (in the UK and Sweden).

The six possible choices in the discrimination task included the target emotion and five distractors. Among those five distractors were four emotions and a “none-of-the-above” option. The “none-of-the-above” option was proposed in accordance with Frank and Stennett (2001) and O’Reilly et al. (2016), to avoid the possibility of agreement artifacts.^6^ The four emotions operating as distractors were carefully selected among the 20 possible emotional states to make the task equally difficult for all target emotions. Lundqvist et al. (2013) were able to create a similarity/dissimilarity matrix for those 20 emotional states. This matrix was established from over 700 participants rating the similarity/dissimilarity of each of the 20 emotions/mental states involved here against all of the other 20 emotions/mental states. Using this matrix, we classified different ranges of similarity (corresponding to very similar, quite similar, quite dissimilar, and very dissimilar) for each target emotion and selected one distractor emotion in each of those ranges (see emotion similarity/dissimilarity matrix in the supplementary material for details). Importantly, each emotion had an equal chance to be selected as a distractor.

In the UK and Sweden, the dimension analysis of each emotional recording included (a) a question about *valence* (“how positive or negative is this emotional expression?”) that was rated between 1 (*very negative*) and 5 (*very positive*), (b) a question about *arousal* (“how strongly does this emotion make you feel?”) that was rated between 1 (*not at all*) and 5 (*very strongly*) and (c) a question about *intensity* (“how intense is this emotional expression?”) that was rated between 1 (*calm*) and 5 (*high intensity*).

### Participants

Altogether, a total of 1,739 complete responses were recorded from the three data collection sites (UK: *n* = 427 [283 females]; Sweden: *n* = 632 [405 females]; Israel: *n* = 461 [309 females]). A minimum of 20 survey-responders/participants completed each survey (per data collection site). The average age of the participants was 38 years (range: 18–90) in the UK, 46 years (range: 17–80) in Sweden, and 32 years in Israel (range: 18–79). Participants were recruited using existing research participant databases and university mailing lists, as well as through online resources.

### Data treatment and analysis

**Fig. 1:**
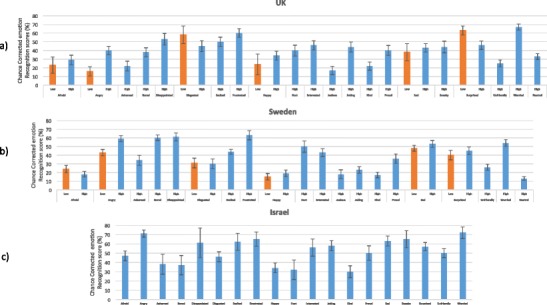
Chance-corrected emotion recognition scores obtained for each emotion and each level of intensity (high, low; when applicable) in the UK (**a**), Sweden (**b**), and Israel (**c**)

A raw recognition rate was computed separately for each individual emotional recording. Given that there were six response options, this score was then adjusted for the chance rate using Cohen’s kappa [chance-corrected recognition rate = (proportion of raw correct – (1/6)/(5/6)], as had been the case in previous work of similar nature (Tottenham et al., 2009, and O’Reilly et al., 2016). When the chance-corrected emotion recognition rates (CCRs) were below 0, they were adjusted to 0. We also calculated whether the target emotion was selected above chance with a binomial test for each stimulus. We report the raw emotion recognition rates, the *p* values for the binomial tests, the CCRs, and the measures of emotional valence, intensity, and arousal (when available) in stimulus item level tables (Table B for the UK, Table C for Sweden, and Table D for Israel) in the supplementary materials. We also report averaged recognition rates (and dimension ratings, when available) across stimuli and respondents for each emotion (and each intensity level, when applicable) and each site in emotion level tables (Table 3 for the UK, Table 4 for Sweden, and Table 5 for Israel; a graphical summary can be found in Fig. 1).^7^ In addition, using Pearson’s correlations, we calculated the intercorrelations between CCRs and ratings of intensity, valence, and arousal for Sweden and the UK overall (Table 6) and for each emotion (Table 7). The data could not be compared across sites due to variation in the experimental conditions, and particularly in the number, age, and sex of the respondents. Finally, we calculated for each site and each emotion category the duration of the emotional voice stimuli (supplementary materials, Table F).

**Table 3:** Summary of the validation data in the UK, including the mean, range, and median of the chance-corrected recognition rates (CCRs), as well as the mean valence, arousal, and intensity for the 20 emotions (and neutral) portrayed by British actors (per intensity of expression, when applicable)

Emotion	Expressed Intensity	N Stimuli	N Respondents	Chance-Corrected Accuracy (%)			Mean Emotional Ratings		
				Mean (SD)	Range	Median	Intensity (SD)	Valence (SD)	Arousal (SD)
Afraid	Low	10	213	23 (30)	0–89	11	3.32 (0.34)	2.19 (0.39)	3.02 (0.30)
	High	30	642	29 (30)	0–94	23	3.46 (0.38)	2.21 (0.40)	3.23 (0.31)
Angry	Low	10	211	16 (17)	0–48	9	3.11 (0.33)	2.45 (0.19)	3.02 (0.37)
	High	35	750	40 (29)	0–95	43	3.56 (0.49)	2.29 (0.32)	3.24 (0.41)
Ashamed	High	29	612	22 (31)	0–88	0	3.13 (0.37)	2.27 (0.29)	3.01 (0.31)
Bored	High	33	704	38 (29)	0–84	40	3.25 (0.35)	2.31 (0.37)	2.99 (0.28)
Disappointed	High	24	518	56 (33)	0–95	58	3.23 (0.43)	2.33 (0.39)	3.06 (0.32)
Disgusted	Low	8	168	58 (29)	0–88	65	3.48 (0.32)	2.19 (0.44)	3.08 (0.32)
	High	29	620	45 (35)	0–94	48	3.51 (0.30)	2.29 (0.50)	3.26 (0.31)
Excited	High	35	747	50 (31)	0–95	54	3.65 (0.35)	3.73 (0.56)	3.37 (0.32)
Frustrated	High	27	578	60 (26)	0–100	60	3.58 (0.45)	2.33 (0.28)	3.19 (0.39)
Happy	Low	4	90	24 (24)	0–53	32	3.00 (0.33)	3.58 (0.27)	2.80 (0.31)
	High	42	890	34 (33)	0–100	22	3.39 (0.43)	3.44 (0.62)	3.12 (0.42)
Hurt	High	26	555	40 (32)	0–94	46	3.33 (0.33)	2.32 (0.32)	3.21 (0.35)
Interested	High	34	724	46 (32)	0–100	47	3.22 (0.38)	3.39 (0.42)	2.93 (0.32)
Jealous	High	25	537	17 (24)	0–71	4	3.50 (0.32)	2.14 (0.29)	3.20 (0.30)
Joking	High	30	633	44 (32)	0–89	46	3.27 (0.36)	3.57 (0.40)	3.04 (0.35)
Kind	High	36	770	22 (29)	0–82	7	3.27 (0.27)	3.57 (0.39)	3.04 (0.30)
Proud	High	35	747	40 (34)	0–100	46	3.16 (0.33)	3.64 (0.43)	3.03 (0.37)
Sad	Low	10	212	38 (31)	0–88	32	3.12 (0.45)	2.41 (0.49)	2.94 (0.46)
	High	31	662	43 (27)	0–90	47	3.08 (0.42)	2.44 (0.41)	2.98 (0.38)
Sneaky	High	23	487	44 (32)	0–100	52	2.92 (0.46)	2.78 (0.36)	2.77 (0.36)
Surprised	Low	12	255	63 (18)	29–82	70	3.22 (0.40)	3.41 (0.47)	2.97 (0.40)
	High	24	516	46 (24)	0–89	49	3.58 (0.41)	3.36 (0.49)	3.29 (0.42)
Unfriendly	High	32	681	25 (23)	0–76	27	3.09 (0.48)	2.41 (0.49)	2.97 (0.49)
Worried	High	28	605	67 (19)	15–94	71	3.55 (0.44)	2.28 (0.26)	3.24 (0.38)
Neutral	High	33	709	33 (20)	0–70	29	2.45 (0.33)	2.72 (0.28)	2.43 (0.33)

SD refers to the standard deviation

**Table 4:** Summary of the validation data in Sweden, including the mean, range, and median of the chance-corrected recognition rates (CCRs), as well as the mean valence, arousal, and intensity for the 19 emotions (and neutral) portrayed by Swedish actors (per intensity of expression, when applicable)

Emotion	Expressed Intensity	N Stimuli	N Respondents	Chance-Corrected Accuracy (%)			Mean Emotional Ratings		
				Mean (SD)	Range	Median	Intensity (SD)	Valence (SD)	Arousal (SD)
Afraid	Low	39	824	24 (27)	0–95	14	2.33 (0.23)	2.85 (0.27)	2.73 (0.27)
	High	47	1,003	18 (23)	0–84	4	2.22 (0.26)	2.90 (0.36)	2.73 (0.35)
Angry	Low	52	1,092	43 (27)	0–100	47	2.22 (0.30)	3.10 (0.38)	2.80 (0.29)
	High	62	1,321	59 (28)	0–100	67	2.20 (0.37)	3.40 (0.55)	3.03 (0.44)
Ashamed	High	29	625	34 (31)	0–84	20	2.36 (0.19)	2.68 (0.26)	2.69 (0.23)
Bored	High	39	841	60 (20)	10–94	66	2.21 (0.27)	2.63 (0.31)	2.54 (0.25)
Disappointed	High	23	484	61 (22)	0–95	64	2.39 (0.22)	2.71 (0.27)	2.61 (0.21)
Disgusted	Low	35	771	31 (33)	0–89	18	2.29 (0.25)	2.82 (0.32)	2.61 (0.29)
	High	46	964	30 (38)	0–100	3	2.33 (0.30)	3.02 (0.32)	2.76 (0.26)
Excited	High	45	1,007	44 (20)	0–84	46	3.38 (0.50)	3.43 (0.39)	2.91 (0.31)
Frustrated	High	22	459	63 (25)	7–94	73	2.25 (0.23)	2.94 (0.54)	2.61 (0.37)
Happy	Low	41	892	15 (24)	0–70	0	3.19 (0.47)	2.94 (0.30)	2.70 (0.30)
	High	52	1,086	19 (28)	0–95	1	3.32 (0.65)	3.19 (0.36)	2.91 (0.28)
Hurt	High	29	606	50 (35)	0–95	64	2.30 (0.23)	2.80 (0.32)	2.81 (0.29)
Interested	High	25	523	43 (23)	0–84	49	3.26 (0.45)	3.05 (0.37)	2.70 (0.30)
Jealous	High	26	553	18 (26)	0–74	0	2.23 (0.27)	2.88 (0.36)	2.67 (0.23)
Joking	High	44	930	23 (26)	0–82	10	3.18 (0.39)	2.95 (0.27)	2.69 (0.26)
Kind	High	44	924	17 (21)	0–76	8	3.34 (0.40)	2.73 (0.32)	2.68 (0.27)
Proud	High	28	609	36 (28)	0–89	35	3.21 (0.43)	2.92 (0.38)	2.69 (0.30)
Sad	Low	51	1,082	48 (25)	0–89	54	2.25 (0.26)	2.73 (0.31)	2.74 (0.30)
	High	48	1,008	53 (27)	0–94	58	2.14 (0.26)	2.88 (0.39)	2.82 (0.32)
Surprised	Low	27	572	40 (28)	0–83	48	3.14 (0.40)	2.90 (0.31)	2.69 (0.25)
	High	37	769	45 (27)	0–90	52	3.22 (0.50)	3.08 (0.36)	2.83 (0.25)
Unfriendly	High	53	1,119	26 (25)	0–88	18	2.27 (0.31)	2.93 (0.35)	2.77 (0.31)
Worried	High	28	614	54 (20)	0–89	56	2.41 (0.21)	2.85 (0.40)	2.67 (0.31)
Neutral	High	39	824	13 (13)	0–49	13	2.53 (0.28)	2.47 (0.26)	2.45 (0.21)

SD refers to the standard deviation

**Table 5:** Summary of the validation data in Israel, including the mean, range, and median of the chance-corrected recognition rates (CCRs) for the 19 emotions portrayed by Israeli actors

Emotion	*N* Stimuli	*N* Respondents	Chance Corrected Accuracy (%)		
			Mean (*SD*)	Range	Median
Afraid	38	797	47 (33)	0–100	50
Angry	41	867	71 (25)	0–100	78
Ashamed	9	197	38 (32)	0–82	45
Bored	10	205	37 (33)	0–90	40
Disappointed	6	123	61 (39)	4–100	72
Disgusted	48	989	46 (37)	0–100	40
Excited	10	225	62 (29)	14–100	67
Frustrated	16	342	65 (31)	0–100	78
Happy	40	832	34 (33)	0–100	23
Hurt	12	256	32 (36)	0–89	19
Interested	16	330	56 (37)	0–100	55
Joking	30	598	58 (29)	0–100	60
Kind	23	473	30 (30)	0–95	24
Proud	18	383	50 (33)	0–100	49
Sad	41	874	63 (35)	0–100	79
Sneaky	13	293	65 (33)	0–100	64
Surprised	42	880	57 (28)	0–100	65
Unfriendly	23	471	50 (23)	2–87	52
Worried	17	362	72 (26)	10–100	75

*SD* refers to the standard deviation

**Table 6:** Summary of intercorrelations between (chance-corrected) recognition scores and valence, arousal, and intensity ratings in (a) the UK and (b) Sweden

a)	UK	Recognition	Intensity	Valence	Arousal
	Recognition		.287^***^	.033	.289^***^
	Intensity			– .001	.836^***^
	Valence				.071
	Arousal				
b)	SWEDEN	Recognition	Intensity	Valence	Arousal
	Recognition		.36^***^	– .10^**^	.35^***^
	Intensity			.22^***^	.79^***^
	Valence				.14^***^
	Arousal				

The tables indicate Pearson’s correlation coefficients and *p* values for significant relationships between the variables: ^*^p < .05, ^**^p < .05, ^***^p < .001

**Table 7:**
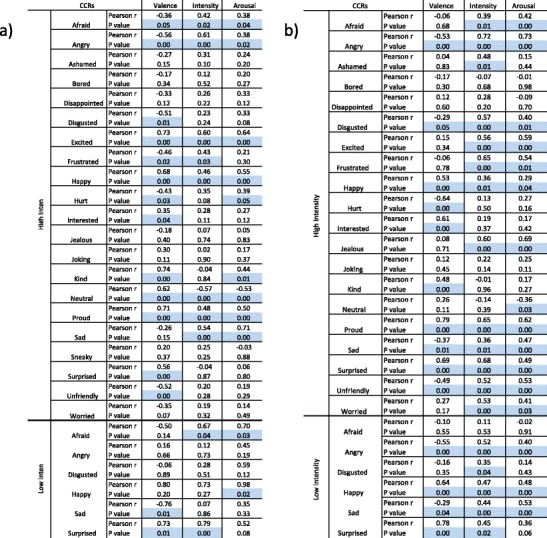
Summary of correlations between (chance-corrected) recognition scores and valence, arousal, and intensity ratings in (a) the UK and (b) Sweden

The tables indicates Pearson’s correlation coefficients and *p* values. Significant *p* values are indicated in light blue (*p* < .05)

## Result and discussion

### UK

An overview of the CCRs and emotional rating scores can be found in Table 3 for each emotion and each intensity level (for basic emotions only). The individual data for each voice stimulus that underwent validation in the UK is available in the supplementary material (Table B). The overall CCR for all emotion categories combined was 39% (*SD*: 31%). This indicates that recognizing an emotion from another’s voice is relatively difficult (as a point of comparison O’Reilly et al., 2016, found a CCR of 63% for recognizing emotions from faces) and variable across stimuli. This variability was apparent both across emotion categories and within an emotion category. Indeed, among the emotions expressed at normal intensity, some were particularly well recognized. This was the case for negative emotions such as *worried* (mean = 67%, *SD* = 19%, median = 71%), *frustrated* (mean = 60%, *SD* = 26%, median = 60%), and *disappointed* (mean = 53%, *SD* = 33%, median = 58%). On the contrary, *kind* (mean = 23%, *SD* = 29%, median = 7%), *ashamed* (mean = 22%, *SD* = 31%, median = 3%), and *jealous* (mean = 17%, *SD* = 24%, median = 4%) had notably low CCRs. Many of the actors who portrayed the UK emotional voices also portrayed (separately) emotions in the face, body, and social context modalities for the EU-Emotion Stimulus Set (O’Reilly et al., 2016). Interestingly, O’Reilly and colleagues also found low CCRs for *kind* and *jealous* expressed through facial emotions (9% and 13%, respectively) and for *jealous* expressed through body language (3%), which was in contrast to the relatively high CCRs when those emotions were represented in social context (61% for *kind* and 44% for *jealous*). This suggests that those emotions are difficult to recognize when simply considering the expressive channels of others and are best recognized in context.

In addition, the CCRs of voice stimuli ranged from 0% to above 80% for most emotions, which indicates that the recognizability of the EU-Emotion voice stimuli within an emotion category was highly variable. As is apparent in Table 3, the effect of expressed intensity is not entirely clear here. Some emotions were recognized better when expressed at low intensity (e.g., *disgusted* and *surprised*), whereas others were recognized better when expressed at high intensity (e.g., *angry* and *happy*). This may be because some emotions (e.g., sadness) are naturally expressed with low-intensity voices, whereas other emotions (e.g., anger) are typically associated with high-intensity voices (e.g., Gopinath, Sheeba, & Nair, 2007), and by producing two levels of intensity per emotions, we may have created congruent and incongruent conditions. The results for the voice stimuli contrast with the results for the EU-Emotion face stimuli, in that those were recognized better at high intensity (O’Reilly et al., 2016). However, these results should be interpreted cautiously, given the smaller number of emotions expressed at low than at high intensity. Nevertheless, as is shown in Table 6a, when all emotions were taken together, CCRs were strongly correlated with ratings of intensity and arousal (themselves correlated) but not with ratings of valence in the UK. This suggests that high perceived intensity and arousal are associated with increased accuracy in recognizing emotions in the vocal modality. However, no correlations between the CCRs and emotional ratings were found for *ashamed*, *bored*, *disappointed*, *jealous*, *sneaky*, and *joking* emotional voices (see Table 7a).

### Sweden

An overview of the CCRs and emotional rating scores can be found in Table 4 for each emotion and each intensity (for basic emotions only). The individual data for each voice stimulus that underwent validation in Sweden are available in the supplementary material (Table C). The overall mean CCR for the Swedish voice stimuli (all emotional categories confounded) was 37% (*SD* = 31%). This approaches the UK overall CCR very closely and confirms the difficulty of recognizing emotions from the voice of others as well as the variation in recognizability of the EU-Emotion voice stimuli. Further exemplifying this variability, Table 4 shows that the CCRs of voice stimuli ranged from 0% to more than 80% within most emotional categories in Sweden. Nevertheless, among emotions expressed at high intensity, *frustrated*, *disappointed*, and *bored* were particularly well recognized (*frustrated*: mean = 63%, *SD* = 25%, median = 73%; *disappointed*: mean = 61%, *SD* = 22%, median = 64%; *bored*: mean = 60%, *SD* = 20%, median = 66%) whereas *jealous*, *kind*, and *neutral* were particularly poorly recognized (*jealous*: mean = 18%, *SD* = 26%, median = 0%; *kind* = mean = 17%, *SD* = 21%, median = 8%; *neutral*: mean = 13%, *SD* = 13%, median = 13%). This pattern of results is similar to that from the UK, in which *frustrated* and *disappointed* were also among the three best-recognized emotions, whereas *jealous* and *kind* among the three worst-recognized emotions.

However, unlike in the UK, the CCRs of basic emotions were not dramatically influenced by their levels of expression, except for *angry* voices, which were recognized better at high than at low intensity (see Table 4). Finally, the correlation analyses showed strong positive correlations between the CCRs and arousal/intensity. This indicates that the higher the perceived intensity/arousal in the voice stimulus, the better the recognition of the expressed emotion in Sweden (see Table 6b). However, no correlations between the CCRs and emotional ratings were found for *bored*, *disappointed*, and *joking* emotional voices (see Table 7b).

### Israel

An overview of the CCRs and emotional rating scores can be found in Table 5 for each emotion expressed at normal intensity. The individual data for each voice stimulus that underwent validation in Israel are available in the supplementary material (Table D). Across all Israeli voice stimuli, the CCR was 53% (*SD* = 33%), which is better than the overall CCRs for the UK and Sweden (means = 39% and 37%, respectively). This might be partly due to the absence of low-intensity emotional recordings in Israel, given that low levels of intensity were associated with lower recognition rates for certain emotions in Sweden and the UK, or due to the much higher initial rejection rate of recordings that were accepted for validation in Israel. It could also be due to the fact that the Hebrew actors portrayed a more spontaneous emotion than the British and Swedish actors, since they were not given the instruction to differentiate two levels of speech intensity. There was as much variability as in the UK and Sweden, though, as is shown by CCRs ranging from 0% to 100% for most emotion categories. However, some emotions were recognized particularly well as categories. This was the case for *angry* (mean = 71%, *SD* = 25%, median = 78%), *frustrated* (mean = 65%, *SD* = 31%, median = 78%), and *worried* (mean = 72%, *SD* = 26%, median = 75%). On the contrary, *kind* (mean = 30%, *SD* = 30%, median = 24%), *hurt* (mean = 32%, *SD* = 36%, median = 19%), and *happy* (mean = 34%, *SD* = 33%, median = 23%) were particularly poorly recognized. This is in accordance with the other sites, where *kind* had a particularly low CCR and *frustrated* had a particularly high CCR, which suggests that the ability to convey those emotions through the voice is stable across sites.

## Discussion

The EU-Emotion Voice Database is a validated collection of 2,159 emotional voice stimuli in three different languages (695 in British English, 1,011 in Swedish, and 453 in Hebrew), which makes it the largest emotional voice database available for scientific use to date. The overall recognition scores for the emotional voice stimuli sets we found (mean CCR: 39% in the UK, 37% in Sweden, and 53% in Israel) were lower than the overall recognition scores reported by some previous emotional voice databases using sentences (means = 70%, 65.4%, and 72.25% in Polzin & Waibel, 1998, when accuracy was determined, respectively by human performance, an emotion acoustic dependent model, and an emotion-dependent suprasegmental model). This is particularly the case for the auditory stimuli depicting the happy and afraid emotional states (happy: 60 or 94% in Polzin & Waibel, 1998 [depending on the computerized model used] and 19%–34% in our study [depending on the site]; afraid: 73% or 60% in Polzin & Waibel, 1998 [depending on the computerized model used] and 18%–47% in our study [depending on the site]).^8^ However, Polzin and Waibel (1998) only had four basic emotional states (happiness, sadness, anger, and fear) and the neutral state. In contrast, we had 20 emotional states (plus neutral), including many complex ones. Our use of numerous complex emotional states could explain our overall lower recognition scores, as those emotional states are typically harder to recognize from a single perceptual channel than are the basic emotional states. For instance, we found that the complex emotions *kind* and *jealous* were among the three most difficult emotions to recognize across all three sites (*kind*: mean CCRs of 22% in the UK, 17% in Sweden, and 30% in Israel; *jealous*: mean CCRs of 17% in the UK and 18% in Sweden^9^). In addition, Banse and Scherer (1996) included 14 emotional states (basic and complex) in their database and found an overall mean recognition score of 48%, which is intermediate between the one we observed (with 20 emotions and neutral) and the one reported by Polzin and Waibel (with only four basic emotions and neutral). This further supports the idea that the decreased mean recognition score observed here in comparison to some emotional voice databases was due to our inclusion of many complex emotions, which are more difficult to recognize than basic ones.

Nevertheless, the overall recognition score we observed here for emotions expressed in the auditory modality was lower than the overall emotion recognition score reported by O’Reilly et al. (2016) for emotions expressed in the visual modality, even though those authors used the same 20 emotional states (mean = 63% for the facial modality, 77% in the bodily modality, and 72% for social scenes), as well as a similar age range and gender distribution for their actors and survey responders. This might suggest that it is harder to recognize emotions from others’ voices than from visual cues, a theory that future studies might investigate further. However, it is noteworthy that the lower emotion recognition scores for stimuli in the auditory modality that we found in the present validation study could also simply be due to our inclusion in the database of voice stimuli with low recognition scores and voice stimuli featuring both semantically emotional sentences and semantically neutral sentences (the latter being relatively hard to produce and recognize). Finally, the emotion stimuli expressed in the visual modality were also longer in duration than the ones expressed in the auditory modality (2–52 s, as opposed to 0.5–4.5 s in the auditory modality).

There is some evidence that vocal bursts convey emotion better than do sentences (mean recognition score of 81% in Scherer, 2000 [ten emotions]; see also Hawk et al., 2009). As a result, and also to avoid the linguistic barriers typically associated with the use of sentences, most contemporary databases have applied emotional intonations to vocal bursts rather than to sentences (see Table 1). In this database, however, sentences were used. This atypical choice was constrained by our need to use our emotional voice stimuli for training purposes (i.e., as part of the educational online game). We believe, though, that it contributes to the high ecological validity of our database. Indeed, prosody is in fact the *melody of speech*, and thus most often is associated with organized speech (sentences) in real life. In addition, the recognition of complex emotions may require stimuli of longer duration than those usually provided through emotional bursts, which we provided through spoken sentences.

Emotion recognition accuracy was variable not only across emotion categories in the EU-Emotion Voice Database, but also within emotion categories. Indeed, within most emotion categories at all three sites, the recognition scores for voice stimuli ranged from 0% to over 80%, which reflects heterogeneity in the recognizability of the voice stimuli included in this database. The stimuli recognized with very little accuracy might, however, be useful for the purpose of machine learning, since, to become more efficient and precise, digital devices need to be trained with emotional stimuli recognized with a good accuracy (as examples that should be recognized by the system), but also with emotional stimuli recognized with poor accuracy (as examples that should not be recognized by the system).

Correlations between the recognition accuracy scores and ratings of valence, arousal, and intensity obtained for each stimulus (in the UK and Sweden) revealed that intensity and arousal were positively associated between themselves and with recognition accuracy, at both sites. Our finding of a positive correlation between ratings of intensity and arousal in the auditory modality are in line with what was found by O’Reilly et al. (2016) in the visual modality, and with the results of Bänziger et al. (2012) across both the visual and auditory modalities, confirming that the arousal and intensity dimensions are strongly associated across modalities. The finding that intensity and arousal correlated with the recognition of emotional voice stimuli is novel, since correlations between recognition scores and ratings of intensity and arousal were not performed as part of previous validation studies in which ratings of both arousal and intensity were collected (Belin et al., 2008; Liu & Pell, 2012), and it suggests that perceived arousal and intensity in the voice could be important cues to the affective state of another. However, it is noteworthy that for certain emotions (e.g., *bored*, *disappointed*, *joking*), the correlations between recognition scores and dimensional ratings (arousal, intensity, valence) were not significant, which suggests that arousal, intensity, and valence are only a crucial factor in emotion recognition accuracy for certain emotions.

To enhance the ecological validity of the emotional vocal stimuli collected for the EU-Emotion Voice Database, we carefully selected professional actors capable of plausibly enacting emotions. Although there might be some differences between the vocal utterances of acted versus experienced emotions (Douglas-Cowie, Campbell, Cowie, & Roach, 2003), we believe that our careful selection of skilled actors (i.e., actors capable of acting in a naturalistic way) minimized those differences. Importantly, to produce the voice stimuli of the EU-Emotion Voice Database, we recruited the largest number of actors ever employed in an emotional voice database (see Table 1), including children and adult actors of both genders. This allowed for increased individual variability in the created emotional voice stimuli, which will be a useful feature for a database training digital devices through machine learning. In addition, this feature might also be important for the experimental study of emotional prosody perception across development, since children could be better at recognizing emotions in peer-aged voice stimuli than in adult voice stimuli. Indeed, although this hypothesis has not yet been tested, in the visual modality, Easter et al. (2005) showed that adolescents were better at recognizing the facial expressions of adolescents than the facial expressions of adults (see also Somerville, Fani, & McClure-Tone, 2011).

The EU-Emotion Voice Database has a number of limitations. First, although emotional voice stimuli were collected in three different languages, a statistical comparison of the validated stimuli across cultures was not possible, due to variation in the experimental parameters across collection sites (i.e., the number of emotions expressed and the intensity levels of expression, number of emotional voice stimuli per emotion category, and number and demographic properties of the participants who participated in the validation). Second, the numbers of stimuli obtained for each of the emotion categories varied within and across sites (*N* stimuli per emotion category expressed at high/normal intensity: 23 to 42 in the UK, 23 to 52 in Sweden, 6 to 42 in Israel), reducing the interpretability of the differences between emotion categories. Finally, to be validated, the emotional voice stimuli were split among a number of surveys in each country (20 in the UK, 30 in Sweden, and 34 in Israel). Each survey thus included only a selection of the emotional voice stimuli. As a result, survey respondents judged only a subset of the emotional stimuli, which means that there was a degree of heterogeneity in respondents between surveys. In addition, in each country the actors did not enact all emotion categories, but only a subset of those emotion categories that depended on which script they received (see the supplementary material, Table A). This was necessary in order to collect the vast amount of data included in the EU-Emotion Voice Database, but it constrained the type and number of possible data analyses. Finally, the emotional voice stimuli were recorded from actors reading scripts, and not from natural emotional speech. As was outlined by Douglas-Cowie et al. (2003), this is a limitation. Indeed, read speech is distinct from spoken speech (Johns-Lewis, 1986), and lacking context can lead actors to express emotion in a caricatured way.

Nevertheless, the EU-Emotional Voice Database will be particularly useful for future studies investigating the perception of emotional prosody, in that it extends previous emotional databases in its number of emotional voice stimuli (2,159), the number and type of emotion categories (20+ neutral), and the number of actors (18); see Table 1. It is also the only emotional voice database to date that has included emotional voice stimuli in three different languages (British English, Swedish, and Hebrew; see Table 1) from both child and adult actors.

Because the EU-Emotion Voice Database matches the number of emotions and expression intensities that are also part of the EU-Emotion Stimulus Set (O’Reilly et al., 2016), the EU-Emotion materials provide a pool of stimuli portraying emotion in the auditory modality that can be used in conjunction with the visual emotional stimuli (i.e., facial expressions, body language, social scenes). This matching of materials will allow for research into cross-modal deficits of emotion recognition that are present in certain clinical conditions (e.g., autism, but also anorexia, depression, or schizophrenia: Golan, Sinai-Gavrilov, & Baron-Cohen, 2015; Hoekert, Kahn, Pijnenborg, & Aleman, 2007; Kan, Mimura, Kamijima, & Kawamura, 2004; Kucharska-Pietura, Nikolaou, Masiak, & Treasure, 2004) and provide the unique possibility to move recognition assessment and expression training beyond basic emotions toward complex and difficult states.

### Author note

The research leading to these results received funding from the European Community’s Seventh Framework Programme (FP7/2007-2013) under Grant Agreement No. 289021 (www.asc-inclusion.eu). S.B. was supported by the Swedish Research Council (Grant No. 523-2009-7054), and S.B.-C. was supported by the Autism Research Trust, the MRC, the Wellcome Trust, and the National Institute for Health Research (NIHR) Collaboration for Leadership in Applied Health Research and Care East of England, at the Cambridgeshire and Peterborough NHS Foundation Trust. The views expressed are those of the author(s) and not necessarily those of the NHS, the NIHR, or the Department of Health.

## Electronic supplementary material

ESM 1 (DOCX 15 kb)

Table A Script A and B listing the sentences said by the actors for each emotional category. In yellow, we indicate the sentences that were semantically congruent with the emotion of interest. (DOCX 34 kb)

Table B Item level summary table of validation data in the UK. Stimuli are ordered from the least to the best recognized for each emotional category. In yellow, we indicate the sentences that were semantically congruent with the emotion of interest. (XLSX 157 kb)

Table C Item level summary table of validation data in Sweden. Stimuli are ordered from the least to the best recognized for each emotional category. In yellow, we indicate the sentences that were semantically congruent with the emotion of interest. (XLSX 215 kb)

Table D Item level summary table of validation data in Israel. Stimuli are ordered from the least to the best recognized for each emotional category. In yellow, we indicate the sentences that were semantically congruent with the emotion of interest. (XLSX 105 kb)

Table E Summary of the validation data including mean, range and median chancecorrected recognition rates (CCR) as well as mean valence, arousal and intensity (when applicable) for all emotions (per intensity of expression when applicable, and separately depending on whether the sentence was semantically meaningful or semantically neutral) for each site (tabulation 1: UK, tabulation 2: Sweden, tabulation 3: Israel). SD refers to standard deviation. (XLSX 65 kb)

Table F Average duration of the emotional voice stimuli per emotion category in the UK (left) and in Sweden (right). SD refers to standard deviation. (XLSX 36 kb)

## Footnotes

1 Freely available to download: www.autismresearchcentre.com/arc_tests.

2 *Social scenes* refer to short videos extracted from popular television series, featuring an emotional state that can only be identified from contextual visual cues (the speech is blurred).

3 Most of the British English speaking actors had also been used in the production of emotional face stimuli and emotional body stimuli, as part of the EU-Emotion Stimulus Set (O’Reilly et al., 2016, Table 1). The same code names was used.

4 *Jealous* and *neutral* were not recorded in Israel.

5 *Sneaky* has two different possible translations in Swedish (*busig* and *lömsk*), and those translations have very different meanings (*busig* approximately corresponding to *rowdy*, *lömsk* corresponding to *sinister*), neither of which is a straight or unambiguous translation of *sneaky*. As a result, the *sneaky* stimuli were not recorded in Sweden.

6 The “none-of-the-above” option was selected at rates of 10% in UK, 15% in Sweden, and 4% in Israel.

7 Table E (tabulation 1–3, supplementary materials) presents the emotion level tables for each site, with stimuli further subdivided on the basis of whether they consisted of semantically neutral or semantically meaningful sentences. Note that, as expected, semantically meaningful sentences are often recognized better than semantically neutral sentences. This is particularly true for certain emotions (e.g., jealousy), indicating that recognizing those emotions may be highly reliant on semantic information.

8 Unfortunately, emotion recognition accuracy scores determined by human performance were not available in Polzin and Waibel (1998) for each individual emotion (happy, afraid, angry, and sad). This slightly undermines the comparison, since our recognition scores, in contrast, were obtained on the basis of human performance.

9 *Jealous* was not recorded in Israel.

## References

[CR1] Abelin, Å., & Allwood, J. (2000, September). *Cross linguistic interpretation of emotional prosody*. Paper presented at the ISCA Tutorial and Research Workshop (ITRW) on Speech and Emotion, Newcastle, Northern Ireland.

[CR2] Asperger H (1944). Die “Autistischen Psychopathen” im Kindesalter. European Archives of Psychiatry and Clinical Neuroscience.

[CR3] Banse R, Scherer KR (1996). Acoustic profiles in vocal emotion expression. Journal of Personality and Social Psychology.

[CR4] Bänziger T, Mortillaro M, Scherer KR (2012). Introducing the Geneva Multimodal expression corpus for experimental research on emotion perception. Emotion.

[CR5] Belin P, Fillion-Bilodeau S, Gosselin F (2008). The Montreal Affective Voices: A validated set of nonverbal affect bursts for research on auditory affective processing. Behavior Research Methods.

[CR6] Douglas-Cowie E, Campbell N, Cowie R, Roach P (2003). Emotional speech: Toward a new generation of databases. Speech Communication.

[CR7] Easter, J., McClure, E. B., Monk, C. S., Dhanani, M., Hodgdon, H., Leibenluft, E., ... Ernst, M. (2005). Emotion recognition deficits in pediatric anxiety disorders: Implications for amygdala research. *Journal of Child & Adolescent Psychopharmacology, 15,* 563–570. 10.1089/cap.2005.15.563 16190788

[CR8] Ekman P, Friesen WV (1971). Constants across cultures in the face and emotion. Journal of Personality and Social Psychology.

[CR9] Fernald, A. (1989). Intonation and communicative intent in mothers’ speech to infants: Is the melody the message? *Child Development*, 1497–1510. 2612255

[CR10] Fernald A, Morikawa H (1993). Common themes and cultural variations in Japanese and American mothers’ speech to infants. Child Development.

[CR11] Frank MG, Stennett J (2001). The forced-choice paradigm and the perception of facial expressions of emotion. Journal of Personality and Social Psychology.

[CR12] Fridenson-Hayo, S., Berggren, S., Lassalle, A., Tal, S., Pigat, D., Bölte, S., ... Golan, O. (2016). Basic and complex emotion recognition in children with autism: Cross-cultural findings. *Molecular Autism, 7*, 52. 10.1186/s13229-016-0113-9 PMC5168820 28018573

[CR13] Globerson E, Amir N, Kishon-Rabin L, Golan O (2015). Prosody recognition in adults with high-functioning autism spectrum disorders: From psychoacoustics to cognition. Autism Research.

[CR14] Golan O, Baron-Cohen S, Hill JJ, Rutherford MD (2007). The “reading the mind in the voice” test—revised: A study of complex emotion recognition in adults with and without autism spectrum conditions. Journal of Autism and Developmental Disorders.

[CR15] Golan O, Sinai-Gavrilov Y, Baron-Cohen S (2015). The Cambridge Mindreading Face–Voice Battery for Children (CAM-C): Complex emotion recognition in children with and without autism spectrum conditions. Molecular Autism.

[CR16] Gopinath, D. P., Sheeba, P. S., & Nair, A. S. (2007, March). *Emotional analysis for Malayalam text to speech synthesis systems*. Paper presented at the International Conference on Electronic Science, Information Technology and Telecommunication-SETIT 2007, Tunisia.

[CR17] Hawk ST, Van Kleef GA, Fischer AH, Van Der Schalk J (2009). “Worth a thousand words”: Absolute and relative decoding of nonlinguistic affect vocalizations. Emotion.

[CR18] Hoekert M, Kahn RS, Pijnenborg M, Aleman A (2007). Impaired recognition and expression of emotional prosody in schizophrenia: Review and meta-analysis. Schizophrenia Research.

[CR19] Johns-Lewis C (1986). *Intonation in discourse*.

[CR20] Kan Y, Mimura M, Kamijima K, Kawamura M (2004). Recognition of emotion from moving facial and prosodic stimuli in depressed patients. Journal of Neurology, Neurosurgery & Psychiatry.

[CR21] Kanner L (1943). Autistic disturbances of affective contact. Nervous Child.

[CR22] Kucharska-Pietura K, Nikolaou V, Masiak M, Treasure J (2004). The recognition of emotion in the faces and voice of anorexia nervosa. International Journal of Eating Disorders.

[CR23] Liu P, Pell MD (2012). Recognizing vocal emotions in Mandarin Chinese: A validated database of Chinese vocal emotional stimuli. Behavior Research Methods.

[CR24] Lundqvist, D., Berggren, S., O’Reilly, H., Tal, S., Fridenson, S., Golan, S., … Bölte, S. (2013, May). *Recognition and expression of emotions in autism: Clinical significance and hierarchy of difficulties perceived by parents and experts*. Paper presented at the 12th Annual International Meeting for Autism Research (IMFAR 2013), International Society for Autism Research (INSAR), San Sebastián, Spain.

[CR25] Marchi, E., Schuller, B., Baron-Cohen, S., Lassalle, A., O’Reilly, H., Pigat, D., … Berggren, S. (2015, March). *Voice emotion games: Language and emotion in the voice of children with autism spectrum condition*. Paper presented at the 3rd International Workshop on Intelligent Digital Games for Empowerment and Inclusion (IDGEI 2015), part of the 20th ACM International Conference on Intelligent User Interfaces, IUI, Atlanta, GA.

[CR26] Niimi, Y., Kasamatsu, M., Nishimoto, T., & Araki, M. (2001, August). *Synthesis of emotional speech using prosodically balanced VCV segments*. Paper presented at the 4th ISCA Tutorial and Research Workshop (ITRW) on Speech Synthesis, Perthshire, Scotland.

[CR27] O’Reilly, H., Pigat, D., Fridenson, S., Berggren, S., Tal, S., Golan, O., … Lundqvist, D. (2016). The EU-Emotion Stimulus Set: A validation study. *Behavior Research Methods, 48,* 567–576. 10.3758/s13428-015-0601-4 10.3758/s13428-015-0601-4 26424443

[CR28] Paul R, Shriberg LD, McSweeny J, Cicchetti D, Klin A, Volkmar F (2005). Relations between prosodic performance and communication and socialization ratings in high functioning speakers with autism spectrum disorders. Journal of Autism and Developmental Disorders.

[CR29] Pell MD, Paulmann S, Dara C, Alasseri A, Kotz SA (2009). Factors in the recognition of vocally expressed emotions: A comparison of four languages. Journal of Phonetics.

[CR30] Pereira, C. (2000, September). *Dimensions of emotional meaning in speech*. Paper presented at the ISCA Tutorial and Research Workshop (ITRW) on Speech and Emotion, Newcastle, Northern Ireland.

[CR31] Polzin, T. S., & Waibel, A. (1998, January). *Detecting emotions in speech*. Paper presented at the Second International Conference on Cooperative Multimodal Communication, CMC 98, Tilburg, The Netherlands.

[CR32] Rutherford MD, Baron-Cohen S, Wheelwright S (2002). Reading the mind in the voice: A study with normal adults and adults with Asperger syndrome and high functioning autism. Journal of Autism and Developmental Disorders.

[CR33] Scherer, K. R. (2000). A cross-cultural investigation of emotion inferences from voice and speech: Implications for speech technology. In *Proceedings of INTERSPEECH 2000* (Vol. 2, pp. 379–382). Beijing, China: ISCA. Retrieved from http://dblp.uni-trier.de/db/conf/interspeech/interspeech2000.html

[CR34] Scherer KR, Ellgring H (2007). Are facial expressions of emotion produced by categorical affect programs or dynamically driven by appraisal?. Emotion.

[CR35] Schröder M (2003). Experimental study of affect bursts. Speech Communication.

[CR36] Somerville LH, Fani N, McClure-Tone EB (2011). Behavioral and neural representation of emotional facial expressions across the lifespan. Developmental Neuropsychology.

[CR37] Tottenham, N., Tanaka, J.W., Leon, A.C., McCarry, T., Nurse, M., Hare, T.A., … Nelson, C. (2009). The NimStim set of facial expressions: judgments from untrained research participants. *Psychiatry Research, 168,* 242–249. 10.1016/j.psychres.2008.05.006 PMC3474329 19564050

[CR38] Van Bourgondien ME, Woods AV, Schopler E, Mesibov GB (1992). Vocational possibilities for high-functioning adults with autism. *High-functioning individuals with autism*.

