# Supplementary material for: The EU-Emotion Voice Database
Source: Behav Res Methods. 2018 Apr 30;51(2):493–506. doi: 10.3758/s13428-018-1048-1 (PMC6478635; doi:10.3758/s13428-018-1048-1)
Supplement: Supplementary file 1 — (DOCX 15 kb) [file 13428_2018_1048_MOESM1_ESM.docx]

**Supplementary data – Emotions matrix.**

The matrix below shows an overview of the emotions used as control emotions 1-4 in the recognition task. Each control emotion was selected based on its similarity/dissimilarity to the target emotion on a 5 point similarity/dissimilarity scale (from Lundqvist et al., 2014) with 1 being dissimilar and 5 being very similar. Control emotions 1-4 were always selected from the same range of dissimilarity, so that control emotion #1 was selected within a similarity range of 2.3 – 2.7 (corresponding to “very similar”), control emotion 2 within 1.9 – 2.2, (corresponding to “quite similar”) control emotion 3 within 1.5 – 1.8 (corresponding to “quite dissimilar”), and control emotion 4 within 1.1 - 1.4 (corresponding to “very dissimilar”).

| ***Target emotion*** | ***Control emotion 1*** | ***Control emotion 2*** | ***Control emotion 3*** | ***Control emotion 4*** | ***Control 5 -  None of the above*** |
| --- | --- | --- | --- | --- | --- |
| **Afraid** | Ashamed | Unfriendly | Disappointed | Kind | None of the above |
| **Angry** | Jealous | Disgusted | Surprised | Happy | None of the above |
| **Ashamed** | Disappointed | Worried | Unfriendly | Proud | None of the above |
| **Bored** | Frustrated | Sad | Hurt | Excited | None of the above |
| **Disappointed** | Worried | Bored | Afraid | Joking | None of the above |
| **Disgusted** | Afraid | Frustrated | Sad | Interested | None of the above |
| **Excited** | Interested | Joking | Hurt | Bored | None of the above |
| **Frustrated** | Sad | Jealous | Sneaky | Kind | None of the above |
| **Happy** | Interested | Surprised | Bored | Angry | None of the above |
| **Hurt** | Worried | Unfriendly | Surprised | Happy | None of the above |
| **Interested** | Excited | Proud | Joking | Disappointed | None of the above |
| **Jealous** | Disappointed | Disgusted | Interested | Kind | None of the above |
| **Joking** | Kind | Interested | Proud | Angry | None of the above |
| **Kind** | Interested | Proud | Excitement | Frustrated | None of the above |
| **Neutral** | Bored | Kind | Surprised | Frustrated | None of the above |
| **Proud** | Excited | Interested | Kind | Afraid | None of the above |
| **Sad** | Afraid | Jealous | Disgusted | Proud | None of the above |
| **Sneaky** | Angry | Disappointed | Ashamed | Kind | None of the above |
| **Surprised** | Happy | Joking | Worried | Bored | None of the above |
| **Unfriendly** | Frustrated | Hurt | Bored | Surprised | None of the above |
| **Worried** | Angry | Disappointed | Disgusted | Happy | None of the above |
