# Supplementary material for: The EU-Emotion Voice Database
Source: Behav Res Methods. 2018 Apr 30;51(2):493–506. doi: 10.3758/s13428-018-1048-1 (PMC6478635; doi:10.3758/s13428-018-1048-1)
Supplement: Supplementary file 2 — Script A and B listing the sentences said by the actors for each emotional category. In yellow, we indicate the sentences that were semantically congruent with the emotion of interest. (DOCX 34 kb) [file 13428_2018_1048_MOESM2_ESM.docx]

| **SCRIPT A** | | | |
| --- | --- | --- | --- |
| **Sentence Code** | **Emotion** | **Intensity level** | **Sentence content** |
| V1A | Happy | High | Hmm, I love chocolate! |
| V1B | Happy | High | It’s wonderful to see you |
| V1C | Happy | High | I knew it from the start |
| V1D | Happy | High | Here we go again |
| V1E | Happy | High | I knew it would happen |
| V1F | Happy | High | What’s all this? |
| V1G | Happy | High | It was always like that |
| V1H | Happy | High | Is this for me? |
| V1I | Happy | High | What’s all this? |
| V2A | Happy | Low | It’s wonderful to see you |
| V2B | Happy | Low | This is my favorite game |
| V2C | Happy | Low | I knew it from the start |
| V2D | Happy | Low | Here we go again |
| V2E | Happy | Low | I knew it would happen |
| V2F | Happy | Low | What’s all this? |
| V2G | Happy | Low | It was always like that |
| V2H | Happy | Low | Is this for me? |
| V2I | Happy | Low | What’s all this? |
| V3A | Afraid | High | I don’t like this |
| V3B | Afraid | High | When will it be over |
| V3C | Afraid | High | It’s always like that – afraid |
| V3D | Afraid | High | I knew it from the start – Afraid |
| V3E | Afraid | High | I knew it would happen – Afraid |
| V3F | Afraid | High | Here we go again – Afraid |
| V3G | Afraid | High | Is this for me? – Afraid |
| V3H | Afraid | High | What’s all this? |
| V4A | Afraid | Low | I don’t like this |
| V4B | Afraid | Low | What was that? |
| V4C | Afraid | Low | It’s always like that – afraid |
| V4D | Afraid | Low | I knew it from the start – Afraid |
| V4E | Afraid | Low | I knew it would happen – Afraid |
| V4F | Afraid | Low | Here we go again – Afraid |
| V4G | Afraid | Low | Is this for me? – Afraid |
| V4H | Afraid | Low | What’s all this? |
| V5A | Disgusted | High | Ugh, cover your mouth when you sneeze! |
| V5B | Disgusted | High | Oh how nasty |
| V5C | Disgusted | High | It was always like that |
| V5D | Disgusted | High | I knew it from the start |
| V5E | Disgusted | High | I knew it would happen |
| V5F | Disgusted | High | What’s all this? |
| V5G | Disgusted | High | Here we go again |
| V5H | Disgusted | High | Is this for me? |
| V6A | Disgusted | Low | Eugh, I can’t look |
| V6B | Disgusted | Low | Oh how nasty |
| V6C | Disgusted | Low | It was always like that |
| V6D | Disgusted | Low | I knew it from the start |
| V6E | Disgusted | Low | I knew it would happen |
| V6F | Disgusted | Low | What’s all this? |
| V6G | Disgusted | Low | Here we go again |
| V6H | Disgusted | Low | Is this for me? |
| V7A | Excited | High | I’m going to bake a Pirate Cake for the children |
| V7B | Excited | High | I can’t wait |
| V7C | Excited | High | What will we do now? |
| V7D | Excited | High | Is it almost over? |
| V7E | Excited | High | Yes, it will happen again |
| V7F | Excited | High | Let me do that |
| V7G | Excited | High | What are you going to do? |
| V8A | Bored | High | There is nothing to do |
| V8B | Bored | High | How much longer! |
| V8C | Bored | High | What will we do now? |
| V8D | Bored | High | Is it almost over? |
| V8E | Bored | High | Yes, it happened again |
| V9A | Disappointed | High | There aren’t any left |
| V9B | Disappointed | High | I tried so hard |
| V9C | Disappointed | High | What will we do now? |
| V9D | Disappointed | High | Yes, it happened again |
| V10A | Hurt | High | That’s not very nice |
| V10B | Hurt | High | How can you say that to me? |
| V10C | Hurt | High | What are you going to do? |
| V10D | Hurt | High | Who told you that? |
| V10E | Hurt | High | Yes, it happened again |
| V11A | Jealous | High | I deserve it more than her |
| V11B | Jealous | High | There’s none left for me |
| V11C | Jealous | High | This doesn’t look good |
| V11D | Jealous | High | Yes, it happened again |
| V11E | Jealous | High | Who told you that? |
| V12A | Joking | High | Made you look! |
| V12B | Joking | High | Try and get it back from me |
| V12C | Joking | High | What will we do now? – Joking |
| V12D | Joking | High | Let me do that – Joking |
| V12E | Joking | High | This doesn’t look good – Joking |
| V12F | Joking | High | What are you going to do? – Joking |
| V12G | Joking | High | Bet you weren’t expecting that |
| V12H | Joking | High | Yes it happened again |
| V13A | Ashamed | High | I shouldn’t have done that |
| V13B | Ashamed | High | That wasn’t very nice of me |
| V13C | Ashamed | High | What are you going to do? |
| V13D | Ashamed | High | Is it almost over? – Ashamed |
| V13E | Ashamed | High | This doesn’t look good – Ashamed |
| V13F | Ashamed | High | Bet you weren’t expecting that |
| V13G | Ashamed | High | This doesn’t look good |

| **SCRIPT B** | | | |
| --- | --- | --- | --- |
| **Sentence Code** | **Emotion** | **Intensity level** | **Sentence content** |
| V15A | Sad | High | I can’t handle this |
| V15B | Sad | High | I’ve lost everything |
| V15C | Sad | High | I knew it from the start |
| V15D | Sad | High | Is this for me? |
| V15E | Sad | High | What's all this? |
| V15F | Sad | High | It was always like that |
| V15G | Sad | High | I knew it would happen |
| V16A | Sad | Low | I’ve lost everything |
| V16B | Sad | Low | I don’t know what I’m going to do now |
| V16C | Sad | Low | I knew it from the start |
| V16D | Sad | Low | Is this for me? |
| V16E | Sad | Low | What's all this? |
| V16F | Sad | Low | It was always like that |
| V16G | Sad | Low | I knew it would happen |
| V17A | Angry | High | What do you think you’re doing? |
| V17B | Angry | High | Look what you’ve done |
| V17C | Angry | High | I knew it from the start |
| V17D | Angry | High | What's all this? |
| V17E | Angry | High | I knew it would happen |
| V17F | Angry | High | It was always like that |
| V17G | Angry | High | Here we go again |
| V17H | Angry | High | Is this for me? |
| V18A | Angry | Low | What do you think you’re doing? |
| V18B | Angry | Low | Look what you’ve done |
| V18C | Angry | Low | I knew it from the start |
| V18D | Angry | Low | What's all this? |
| V18E | Angry | Low | I knew it would happen |
| V18F | Angry | Low | It was always like that |
| V18G | Angry | Low | Here we go again |
| V18H | Angry | Low | Is this for me? |
| V19A | Surprised | High | I didn’t expect to see you here! |
| V19B | Surprised | High | Did that really happen? |
| V19C | Surprised | High | Here we go again –Surprised |
| V19D | Surprised | High | It was always like that |
| V19E | Surprised | High | What's all this? |
| V19F | Surprised | High | Is this for me? |
| V20A | Surprised | Low | Oh my goodness |
| V20B | Surprised | Low | I didn’t expect to see you here! |
| V20C | Surprised | Low | Here we go again –Surprised |
| V20D | Surprised | Low | It was always like that |
| V20E | Surprised | Low | What's all this? |
| V20F | Surprised | Low | Is this for me? |
| V21A | Interested | High | What have you got there? |
| V21B | Interested | High | What happened after that? |
| V21C | Interested | High | What will we do now? |
| V21D | Interested | High | Let me do that |
| V21E | Interested | High | Who told you that? |
| V21F | Interested | High | This doesn't look good |
| V21G | Interested | High | Bet you weren't expecting that |
| V22A | Worried | High | I think I’ve lost it |
| V22B | Worried | High | Oh no, I think its broken! |
| V22C | Worried | High | This doesn't look good |
| V22D | Worried | High | What will we do now? |
| V23A | Frustrated | High | Why won’t this work?! |
| V23B | Frustrated | High | What will we do now? |
| V23C | Frustrated | High | Is it almost over? |
| V23D | Frustrated | High | This doesn't look good |
| V24A | Kind | High | Let me help you |
| V24B | Kind | High | I thought you would like it |
| V24C | Kind | High | What are you going to do? |
| V24D | Kind | High | What will we do now? |
| V24E | Kind | High | Yes it happened again |
| V24F | Kind | High | Is it almost over? |
| V24G | Kind | High | Let me do that |
| V24H | Kind | High | Bet you weren't expecting that |
| V24I | Kind | High | What are you going to do? |
| V25A | Unfriendly | High | What are you doing here? |
| V25B | Unfriendly | High | I can do whatever I want |
| V25C | Unfriendly | High | Is it almost over? |
| V25D | Unfriendly | High | What are you going to do? |
| V25E | Unfriendly | High | Let me do that |
| V25F | Unfriendly | High | What will we do now? |
| V25G | Unfriendly | High | This doesn't look good |
| V26A | Sneaky | High | I’ll do it when nobody is watching |
| V26B | Sneaky | High | Nobody will ever find it here |
| V26C | Sneaky | High | What are you going to do? |
| V26D | Sneaky | High | Who told you that? |
| V26E | Sneaky | High | What will we do now? |
| V26F | Sneaky | High | This doesn't look good |
| V27A | Proud | High | I worked hard on this |
| V27B | Proud | High | I knew I could do it |
| V27C | Proud | High | Yes it happened again |
| V27D | Proud | High | What will we do now? |
| V28D | Proud | High | Let me do that |
| V29D | Proud | High | Bet you weren't expecting that |
| V14A | Neutral/Relaxed | High | Is this for me? |
| V14B | Neutral/Relaxed | High | I knew it from the start |
| V14C | Neutral/Relaxed | High | Here we go again |
| V14D | Neutral/Relaxed | High | I knew it would happen |
| V14E | Neutral/Relaxed | High | What's all this? |
| V14F | Neutral/Relaxed | High | It was always like that |
| V14G | Neutral/Relaxed | High | This doesn't look good |

Extra voices scripts by Actor A

| **Sentence Code** | **Emotion** | **Intensity level** | **Sentence content** |
| --- | --- | --- | --- |
| A-V13H | Ashamed | High | I didn’t mean to hurt anybody’s feelings |
| A-V13I | Ashamed | High | I don’t know what to say |
| A-V9E | Disappointed | High | You’ve done it again |
| A-V21H | Interested | High | I wonder, why?! |
| A-V11F | Jealous | High | It happened again |
| A-V11G | Jealous | High | I knew it from the start |
| A-V27E | Proud | High | All the hard work paid off in the end |
| A-V27F | Proud | High | You’ve done really well |
| A-V26G | Sneaky | High | You didn’t see me |
| A-V22E | Worried | High | Let’s go! |
| A-V22F | Worried | High | I can’t find it anywhere |
| A-V22G | Worried | High | Oh No, this doesn’t look good |

Extra voice scripts Actor B

| **Sentence Code** | **Emotion** | **Intensity level** | **Sentence content** |
| --- | --- | --- | --- |
| B-V17I | Angry | High | Who has just taken the chocolate I put down here |
| B-V8F | Bored | High | Let’s go |
| B-V5I | Disgusted | High | Eugh what’s that smell |
| B-V7H | Excited | High | It’s going to be great |
| B-V25H | Unfriendly | High | I don’t think so |
| B-V25I | Unfriendly | High | You’re too slow |
| B-V25J | Unfriendly | High | I don’t want to talk to you |
| B-V23E | Frustrated | High | I didn’t think this would be so complicated |
| B-V14H | Neutral | High | What are you going to do |
| B-V15H | Sad | High | I’ll miss you |
| B-V15I | Sad | High | I don’t know what to do |
| B-V19G | Surprised | High | I wasn’t expecting that |
| B-V19H | Surprised | High | What are you doing here |
